# Oral semaglutide vs DPP-4 inhibitors in reducing risk of cognitive impairment in elderly patients with HFpEF and obesity

**DOI:** 10.1007/s11739-026-04499-4

**Published:** 2026-08-20

**Authors:** Giuseppe Armentaro, Cristiana Vitale, Velia Cassano, Marilisa Panza, Ilaria Gareri, Maria Mirabelli, Elena Succurro, Francesco Andreozzi, Antonio Brunetti, Gianluigi Savarese, Maurizio Volterrani, Giorgio Sesti, Giuseppe Massimo Claudio Rosano, Angela Sciacqua

**Affiliations:** 1 Geriatrics Division, “Renato Dulbecco” University Hospital of Catanzaro, 88100 Catanzaro, Italy; 2 Department of Human Sciences and Promotion of Quality of Life, San Raffaele Open, University of Rome, Rome, Italy; 3 IRCCS San Raffaele, Rome, Italy; 4 Department of Medical and Surgical Sciences, University Magna Græcia of Catanzaro, 88100 Catanzaro, Italy; 5 Department of Health Sciences, University “Magna Græcia” of Catanzaro, 88100 Catanzaro, Italy; 6 Research Center for the Prevention and Treatment of Metabolic Diseases (CR‑METDIS), University Magna Græcia, 88100 Catanzaro, Italy; 7 Division of Cardiology, Department of Medicine, Karolinska Institutet, and Heart and Vascular and Neuro Theme, Karolinska University Hospital, Stockholm, Sweden; 8 Department of Clinical and Molecular Medicine, University of Rome-Sapienza, 00185 Rome, Italy

**Keywords:** Elderly, HFpEF, Type 2 diabetes, Obesity, Cognitive impairment

## Abstract

Heart failure with preserved ejection fraction (HFpEF), obesity, type 2 diabetes mellitus are common in elderly patients and are associated with an increased risk of cognitive impairment (CoI). In these patients, the protective metabolic and vascular effects of glucagon-like peptide−1 receptor agonists (GLP-1 RAs) are well established. The aim of this study was to evaluate effects of one-year treatment with oral semaglutide versus DPP-4 inhibitors (DPP4i), as an add-on to the hypoglycaemic therapy, on the incidence of CoI in patients with HFpEF, obesity, and type 2 diabetes. 246 elderly outpatients were recruited and divided into two groups (oral semaglutide vs. DPP4i), accurately matched for age, sex, and body mass index (BMI). At one-year follow-up, 106 new cases of CoI were observed in the entire study population (43.1 events/100 patients/year). A significant difference was found between the two groups: 33 cases with oral semaglutide (26.8 events/100 patients/year) versus 73 cases with DPP4i (59.3 events/100 patients/year), p<0.0001. Logistic regression analysis indicated a 77% reduced risk of incident CoI with oral semaglutide compared to DPP4i. In elderly patients with HFpEF, obesity and type 2 diabetes, oral semaglutide seems to have a positive effect in reducing the incidence of cognitive impairment.

**Graphical Abstract:**

**Figure:**
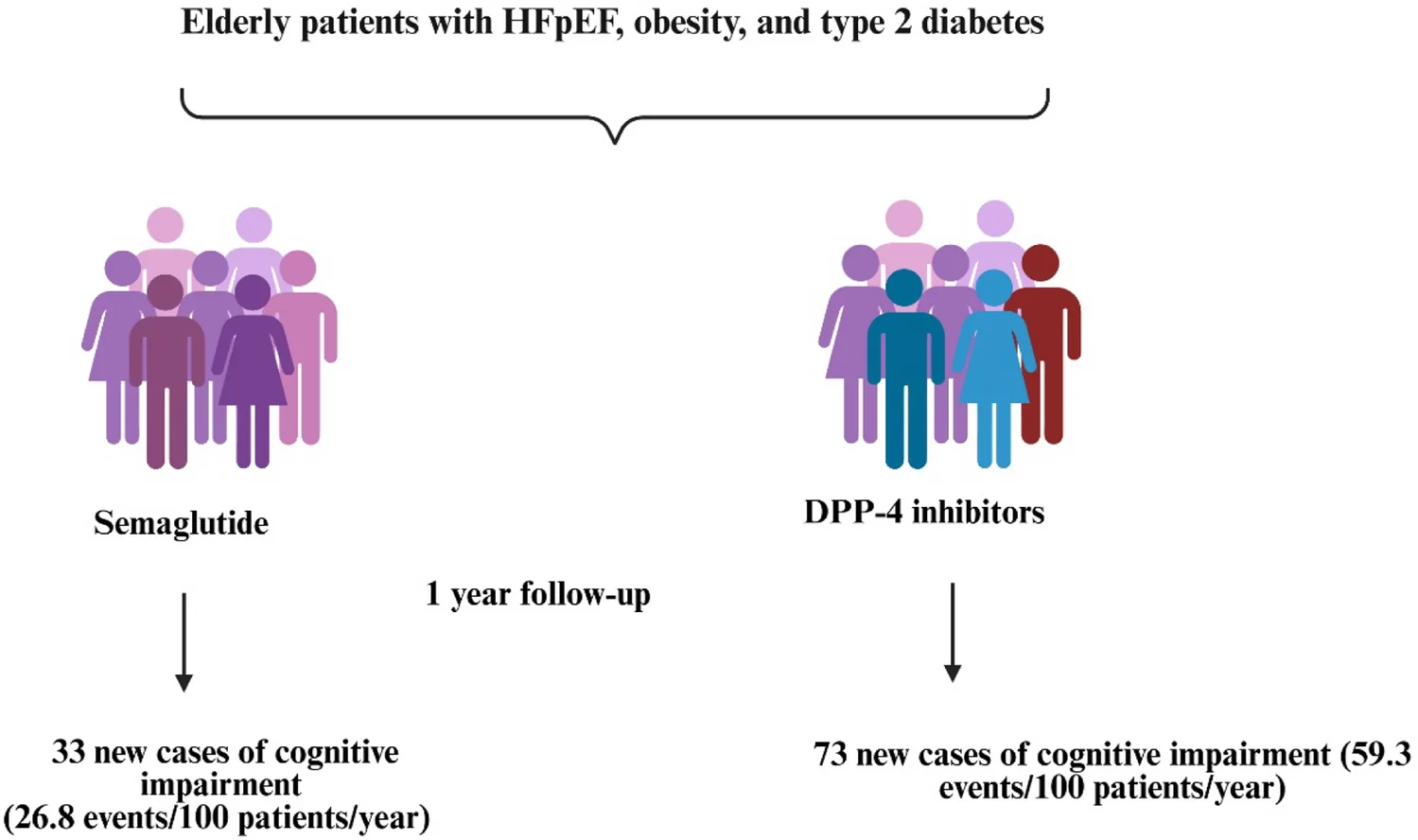

**Supplementary Information:**

The online version contains supplementary material available at https://doi.org/10.1007/s11739-026-04499-4.

## Introduction

Heart failure with preserved ejection fraction (HFpEF) is the most common phenotype of heart failure (HF) in the elderly population. It coexists with several non-cardiovascular comorbidities, including type 2 diabetes mellitus and obesity. These conditions share common risk factors and pathogenic mechanisms [1, 2]. Elderly patients with HFpEF and type 2 diabetes have a higher risk of developing cognitive impairment (CoI), which can be detected in up to 31% of these patients [3, 4].

A meta-analysis of 144 prospective studies showed that type 2 diabetes increases the risk of mild cognitive impairment (MCI) by 1.49-fold. Several mechanisms, such as chronic hyperglycaemia, increased oxidative stress and neuro-inflammation, advanced glycation end-products accumulating in cerebral tissues, impaired mitochondrial function, altered neurotransmitter regulation, compromised blood–brain barrier integrity, cerebral amyloid angiopathy, and dysregulated glucose metabolism in the brain, can collectively compromise neuronal function and contribute to cognitive decline amongst diabetic patients. Moreover, chronic hyperglycemia can promote β-amyloid precursor protein cleavage by β-secretases, favoring the production of the β-amyloid fragment, which accumulates into β-amyloid plaques and simultaneously the hyperphosphorylation of tau protein, leading to the formation of insoluble and cytotoxic tau aggregates [5–7]. Persistently high blood glucose levels in type 2 diabetes may also alter blood–brain barrier integrity and function. This, combined with impaired cerebral glucose metabolism, leads to reduced expression of brain-derived neurotrophic factor (BDNF), which is important for synaptic plasticity and neuroprotection [8].

In addition to these mechanisms, insulin resistance plays a key role in the onset of CoI, causing overproduction of reactive oxygen species (ROS) and the accumulation of β-amyloid in the brain [9]. Both insulin resistance and HFpEF risk increase progressively with higher BMI, and obese elderly patients with HFpEF exhibit lower cognitive performance compared to their non-obese counterparts [10, 11].

To date, only sodium-glucose cotransporter-2 inhibitors (SGLT2i) dapagliflozin and empagliflozin, and with lesser evidence mineralocorticoid receptor antagonists (MRAs) have shown to improve HFpEF patients’ prognosis, regardless of the presence of type 2 diabetes [12–14]. Furthermore, SGLT2i seems to improve cognitive function, depressive symptoms, and functional abilities, regardless of changes in ejection fraction [8, 15–17].

In addition to SGLT2i, glucagon-like peptide−1 receptor agonists (GLP1-RAs), reduce cardiovascular (CV) risk in high-risk patients with type 2 diabetes and/or overweight/obesity [18, 19].

Recent studies have suggested that injectable GLP1-RAs can improve CoI symptoms in patients with type 2 diabetes through several potential mechanisms, including reduced brain insulin resistance, improved brain glucose hypometabolism, and ameliorated oxidative stress [20–22].

However, to date, no study has demonstrated the efficacy of oral semaglutide therapy in reducing the risk of developing CoI in elderly obese patients with HFpEF and type 2 diabetes. Furthermore, considering that GLP1-RAs and DPP4is both act on the incretin system, they offer very different efficacy, cardiovascular benefits and body weight results, the aim of this study was to assess the potential impact of oral semaglutide, compared to dipeptidyl-peptidase−4 inhibitors (DPP-4i) on the incidence of CoI in a cohort of elderly diabetic patients with HFpEF, already under stable treatment with SGLT2i for at least six months.

## Methods

The present study included outpatients with HF from two observational registries. In particular, in this retrospective analysis, 246 patients with HFpEF and type 2 diabetes who started either oral semaglutide or DPP-4 inhibitors (any agent in the class, except saxagliptin) were selected from the MAGIC-HF and CATAMERI registries [15, 16]. All participants provided written informed consent. All participants were over 65 years of age (mean age 79.5 ± 7.7 years) and had been on metformin, statins, and SGLT2i therapy for a minimum of six months prior to initiating oral semaglutide or DPP-4i therapy, as per the inclusion criteria.

All patients underwent full clinical assessment including the evaluation of BMI, comprehensive geriatric assessment (CGA), including the determination of cognitive function using the Montreal Cognitive Assessment (MoCA) score, echocardiogram, and laboratory tests at baseline and one-year follow-up. Quality of life was assessed using the Kansas City Cardiomyopathy Questionnaire – Clinical Summary score (KCCQ-CSS) [23].

All laboratory measurements were performed on peripheral blood samples after at least 12 h of fasting. Serum Albumin was measured with a colorimetric spectrophotometric method (Bromocresol green). Glycaemia was determined by the glucose oxidase method (glucose analyzer, BeckmanCoulter, Milan) and the homeostasis model assessment (HOMA) index was used for the determination of Insulin resistance [24]. Enzymatic methods (Roche Diagnostics GmbH, Mannheim, Germany) were used for determination of blood levels of total cholesterol, low-density lipoprotein (LDL) cholesterol, highdensity lipoprotein (HDL) cholesterol, and triglycerides. Alanine aminotransferase (ALT), aspartate aminotransferase (AST) by pyridoxal phosphate activated (liquid reagent), and gamma-glutamyltransferase (γ-GT) were evaluated by standardized method (COBAS Integra 800-Roche Diagnostics GmbH, Mannheim, Germany). Creatinine was measured using the Jaffé method. The CKD-EPI (Chronic Kidney Disease Epidemiology Collaboration) equation was used for the estimation of glomerular filtration rate (eGFR) [25]. Serum uric acid (UA) levels were assessed using URICASE/POD method (Boehringer Mannheim, Mannheim, Germany). The immuno-turbidimetric method automated system (Cardio Phase hs-CRP, Milan, Italy) was used to assess the high sensitivity C-reactive protein (hs-CRP).

Inclusion criteria for the study were: (1) the presence of HFpEF, (2) Obesity, (3) type 2 diabetes, and (4) age >65 years. Exclusion criteria were: (1) major depressive or psychiatric disorders, (2) dementia, (3) severe liver or kidney failure, (4) use of saxagliptin, (5) insulin therapy and (6) recent (< 3 months) hospitalization or cardiovascular events preceding the initiation of oral semaglutide or DPP-4i therapy.

The study population was stratified into two groups according to the initiation of antidiabetic therapy: oral semaglutide or DPP-4i. The primary study endpoint was the incidence of cognitive impairment (CoI), assessed as a score of <26 points on the MoCA at the one-year follow-up.

### Statistical analysis

After testing for normality, continuous data were expressed as mean ± standard deviation or as median and interquartile range, as appropriate.

To compare study variables between the two groups, the Mann–Whitney test, Student's t-test for unpaired data, and the chi-square test were used when appropriate. A multivariate stepwise logistic regression model was generated to assess the associations between the variables of interest and the incidence of cognitive impairment (CoI), defined as a MoCA score below 26 points at one-year follow-up, with CoI as the dependent variable. The analysis was corrected for variables that differed statistically significantly between the two groups and for variables that could be patho-physiologically associated with the occurrence of CoI. In the multivariable logistic regression model, adjustment was performed for the following baseline covariates: female sex, smoking status, chronic kidney disease (CKD), sleep apnea syndrome (SAS), treatment with β-blockers, PCSK9 inhibitors, antiplatelet therapy, serum albumin, left ventricular ejection fraction (LVEF), and oral semaglutide use. Age was entered into the model per 10-year increase. In addition, changes from baseline to follow-up (Δ) were included for BMI, KCCQ-CSS, HOMA, glycated hemoglobin (HbA1c), and hs-CRP. Odds ratios (OR) for the incidence of CoI were calculated for each independent variable, first identified using a univariate logistic approach, and then included in the multivariate logistic regression model. Statistical analysis was performed using SPSS V20.0 for Windows (SPSS Inc., Chicago, Illinois, USA). Statistical significance was set at *p* < 0.05 for each analysis.

## Results

From a total population of 3245 patients in the MAGIC-HF and CATAMERI registries, 246 patients were identified for inclusion in one of the two treatment groups (oral semaglutide vs. DPP-4 inhibitors). Enrolled patients had a mean age of 79.5 ± 7.7 years, with 168 males and 78 females. The mean MoCA score for the entire population was 26.5 ± 1.6 points, with no statistically significant differences between the two groups. The two groups were similar in terms of age, sex distribution, BMI, and major comorbidities, except for the prevalence of CKD, which was higher in the DPP4i group, and obstructive sleep apnea syndrome, which was more prevalent in the oral semaglutide group (Supplementary Table S1).

In the oral semaglutide group, a greater use of beta-blockers and antiplatelet agents was observed compared to the DPP4i group; in contrast, a higher utilization of proprotein convertase subtilisin/kexin type 9 inhibitors (PCSK9i) was seen in the DPP4i group compared to the oral semaglutide group (Table 1).

**Table 1:** Echocardiographic parameters and pharmacotherapy of study population at baseline according to therapy with oral semaglutide or DPP4i

	All population (n. 246)	Oral semaglutide (n. 123)	DPP4i (n. 123)	*p*^a^
Age, years	79.5 ± 7.7	79.7 ± 9.4	79.3 ± 5.6	0.671^**b**^
Female sex, n (%)	78 (31.7)	32 (26.0)	46 (37.4)	0.055^**a**^
BMI, Kg/m^2^	37.2 ± 4.4	37.1 ± 4.6	37.3 ± 4.3	0.769^**b**^
Arterial hypertension, n(%)	206 (83.7)	106 (86.2)	100 (81.3)	0.299°
AF, n (%)	39 (15.8)	13 (10.6)	26 (21.1)	0.232^a^
Dislipidemia, n (%)	208 (85)	104 (84.5)	104 (85.4)	0.999^a^
SAS, n (%)	109 (44.3)	74 (60.1)	35 (28.4)	<0.0001^a^
CKD, n (%)	108 (43.9)	28 (22.7)	80 (65.0)	<0.0001^a^
COPD, n (%)	53 (21.5)	24 (19.5)	29 (23.6)	0.438^a^
NAFLD, n (%)	121 (49.2)	58 (47.15)	63 (51.2)	0.524^a^
Smokers, n (%)	51 (20.7)	14 (11.4)	37 (30.1)	0.626^a^
Ex Smokers, n (%)	111 (45.1)	60 (48.8)	51 (41.5)	0.249^a^
KCCQ-CCS, pt	59.91 ± 6.4	59.41 ± 6.7	60.41 ± 6.1	0.224^**b**^
GLS, %	−17.5 ± 1.1	−17.5 ± 1.3	−17.6 ± 0.9	0.387^**b**^
LVEF, %	53.4 ± 2.2	54.2 ± 1.5	53.7 ± 2.7	0.037^**b**^
E/A	0.76 ± 0.13	0.77 ± 0.15	0.76 ± 0.13	0.612^**b**^
E/e’	13.7 ± 2.2	13.6 ± 2.8	13.8 ± 1.6	0.601^**b**^
TAPSE, mm	19.8 ± 2.4	19.6 ± 2.9	19.9 ± 1.9	0.270^**b**^
s-PAP, mmHg	37.9 ± 5.0	37.8 ± 5.2	38.1 ± 4.9	0.687^**b**^
TAPSE/s-PAP, mm/mmHg	0.53 ± 0.11	0.53 ± 0.14	0.53 ± 0.09	0.959^**b**^
IVC, mm	17.8 ± 1.4	17.6 ± 1.5	17.9 ± 1.3	0.059^b^
β-blockers, n (%)	103 (41.8)	80 (65.0)	23 (18.7)	<0.0001^a^
ACEi/ARBs, n (%)	206 (83.7)	106 (86.2)	100 (81.3)	0.299^a^
MRAs, n (%)	120 (48.8)	58 (47.1)	62 (50.4)	0.609^a^
CCBs, n (%)	109 (44.3)	52 (42.3)	57 (46.3)	0.521^a^
SGLT2i, n (%)	246 (100)	123 (100)	123 (100)	0.999^a^
Loop diuretics, n (%)	201 (81.7)	103 (83.7)	98 (79.6)	0.409^a^
Metformin, n (%)	246 (100)	123 (100)	123 (100)	0.999^a^
NOACs, n (%)	29 (11.8)	10 (8.13)	19 (15.4)	0.751^a^
VKAs, n (%)	11 (4.5)	3 (2.43)	8 (6.5)	0.122^a^
Anti PLT, n (%)	117 (47.5)	77 (62.6)	44 (35.7)	<0.0001^a^
Ezetemibe, n (%)	116 (47.1)	54 (43.9)	62 (50.4)	0.307^a^
Statins, n (%)	246 (100)	123 (100)	123 (100)	0.999^a^
PCSK9i, n (%)	60 (24.4)	16 (13.0)	44 (35.8)	<0.0001^a^

^a^Performed by χ-square test

^b^Performed by t-test for unpaired data

*DDP4i* Dipeptidyl peptidase−4 inhibitors, *LVEDV/BSA* left ventricular end-diastolic volume/body surface area, *LVESV/BSA* left ventricular end-sistolic volume/body surface area, *GLS* Global longitudinal strain, *GWE* global myocardial work efficiency, *LAVi* left atrial volme index, *LVEF* left ventricular ejection fraction, *CI* Cardiac Index, *RVOTp* Right Ventricular Outflow Tract proximal, *E/A* ratio between wave E (the wave of rapid filling in early diastole) and wave A (the wave of atrial contraction), *E/e’* between wave E and wave e′ (reliable estimate of changes in end-diastolic blood pressure), *TAPSE* Tricuspid annular plane systolic excursion, *s-PAP* systolic pulmonary arterial pressure *ACEi* Angiotensin-converting enzyme inhibitors *ARBs* Angiotensin II receptor blockers, *MRAs* mineralocorticoid receptor antagonists, *CCBs* Calcium channel blockers, *ARNI* angiotensin receptor/neprilysin inhibitor *SGLT2i* sodium-glucose cotransporter 2 inhibitors, *NOACs* Non-vitamin K oral anticoagulants, *VKAs* Vitamin K antagonists, *PLTs* Platelets, *PCSK9i* proprotein convertase subtilisin/kexin type 9 inhibitors

At baseline, all patients in the semaglutide group started with the lowest dose of oral semaglutide (3 mg/day). At the 12-month follow-up, 22% of patients remained on the lowest dose (3 mg/day), 25.2% were on the intermediate dose (7 mg/day), and 65% were on the highest dose (14 mg/day). During the study period only 7 episodes of nausea and 2 episodes of diarrhea were reported in the semaglutide group, none of which required discontinuation of the drug.

At one year after the initiation of oral semaglutide or DPP4i therapy, there was a significant reduction in BMI: − 1.5 (− 3.3/−0.6) kg/m^2^ in the entire population. This change was more pronounced in the oral semaglutide group: − 2.9 (− 4.3/−1.5) kg/m^2^, compared to − 0.7 (− 1.4/0.0) kg/m^2^ in the DPP4i group (*p* < 0.0001). Across the entire study population, we observed a significant reduction in BMI from 37.2 ± 4.4 to 35.5 ± 4.5, *p* < 0.0001; specifically, in the oral semaglutide group from 37.1 ± 4.6 to 34.1 ± 4.5, *p* < 0.0001, and in the DPP4i therapy group from 37.3 ± 4.3 to 36.9 ± 4.0, *p* = 0.141.

Weight loss was associated with a significant improvement in insulin sensitivity in the entire population, as indicated by reductions in HOMA: − 1.1 (− 0.8/−1.7), with a more pronounced reduction in the oral semaglutide group: − 1.7 (− 2.2/−1.1), compared to the DPP4i group: − 0.8 (− 1.2/0.4), (*p* < 0.0001). There was also a significant reduction in hs-CRP levels: − 0.9 (− 1.5/−0.4) mg/l, which was significantly greater in the oral semaglutide group: − 1.3 (− 1.9/−0.7) mg/l, compared to the DPP4i group: − 0.6 (− 1.0/−0.3) mg/l (*p* < 0.0001).

Furthermore, across the entire study population, we observed significant variations in systolic blood pressure values from 141.8 ± 8.3 to 130.7 ± 6.8, *p* < 0.0001, but not in diastolic blood pressure from 84.1 ± 3.0 to 83.8 ± 3.0, p = 0.227. From a metabolic point of view, we also observed significant improvements, such as blood glucose from 141.6 ± 8.2 to 130.7 ± 6.7, p<0.0001, total cholesterol from 162.1 ± 11.6 to 149.4 ± 8.6, *p* < 0.0001, HDL from 39.7 +-3.1 to 42.4+-2.8, p<0.0001, triglycerides from 155.6 ± 8.5 to 130.7130.7 ± 9.4, *p* < 0.0001.

Of particular interest, an improvement in heart failure (HF) symptoms was observed in the oral semaglutide group, with positive changes in the Kansas City Cardiomyopathy Questionnaire – Clinical Summary (KCCQ-CS) score: 8.0 (2.0/14.0), whereas a worsening was observed in the DPP4i group: − 3.0 (− 8.0/2.0), (*p* < 0.0001) (Table 2).

**Table 2:** Incidence of CoI in the study population and variation of the main study variables at 1 year of follow-up in the two treatment groups

All Population (n. 246)	Baseline (*n* = 246)	Follow-up (* n* = 246)	*p*
MoCA, pt	26.5 ± 1.6	25.9 ± 1.8	<0.0001
HOMA, pt	7.8 ± 0.9	6.5 ± 0.7	<0.0001
hs-CRP, mg/l	4.1 ± 0.6	3.1 ± 0.6	<0.0001
BMI, Kg/m^2^	37.2 ± 4.5	35.5 ± 4.5	<0.0001
KCCQ-CSS, pt	59.9 ± 6.4	62.3 ± 7.2	<0.0001
HbA1c, %	8.1 ± 0.6	7.4 ± 0.5	<0.0001

	All Population (*n* = 246)	Oral semaglutide (*n* = 123)	DPP4i (*n* = 123)	*p*
Cognitive impairment, n (events/100 patients/years)	106 (43.1)	33 (26.8)	73 (59.3)	<0.001^a^
Δ MoCA, pt	0.0 (− 2.0/1.0)	0.0 (− 1.0/1.0)	−1.0 (− 3.0/0.0)	<0.0001^b^
Δ HOMA, pt	−1.1 (− 0.8/−1.7)	−1.7 (− 2.2/1.1)	−0.8 (1.2/0.4)	<0.0001^b^
Δ hs-CRP, mg/l	−0.9 (− 1.5/−0.4)	−1.3 (− 1.9/−0.7)	−0.6 (− 1.0/−0.3)	<0.0001^b^
Δ BMI, Kg/m^2^	−1.5 (− 3.3/−0.6)	−2.9 (− 4.3/−1.5)	−0.7(− 1.4/0.0)	<0.0001^b^
Δ KCCQ-CSS, pt	2.0 (− 4.0/9.0)	8.0 (2.0/14.0)	−3.0 (− 8.0/2.0)	<0.0001^b^
Δ HbA1c, %	−0.5 (− 0.9/−0.3)	−0.8 (− 1.4/−0.4)	−0.4 (− 0.5/−0.2)	<0.0001^b^

*Δ* variation between baseline and 1-year follow-up, *DPP4i* Dipeptidyl peptidase−4 inhibitors, *MoCA* Montreal Cognitive Assessment, *HOMA* Homeostasis model assessment, *hs-CRP* high sensitivity C reactive protein, *BMI* Body mass index, *KCCQ-CSS* Kansas City Cardiomyopathy Questionnaire- clinical summary score *HbA1c* glycated haemoglobin

^a^Performed by χ-square test

^b^Performed by mann whitney test

**Fig. 1:**
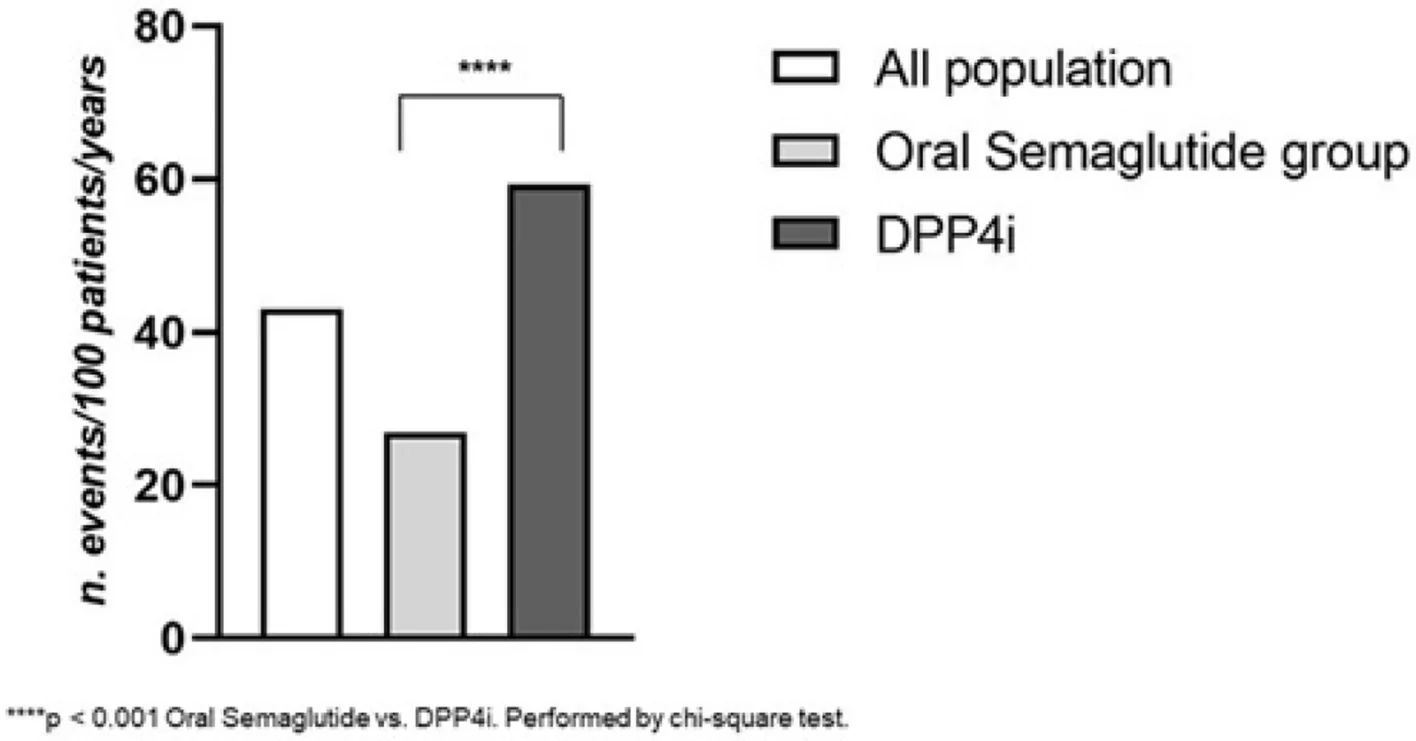
Incidence of CoI in the study population at one-year follow-up. *CoI* Cognitive Impairment, *DPP4i* Dipeptidyl peptidase−4 inhibitors;

In the entire study population, 106 (43.1%) new cases of cognitive impairment (CoI) were detected at the one-year follow-up, with a significantly lower percentage of cases in the oral semaglutide group: 33 (26.8 events/100 patients/years) vs. 73 (59.3 events/100 patients/years) in the DPP4i group (*p* < 0.001) (Table 2) (Fig. 1). In addition, a greater decline in MoCA score was observed in the group receiving DPP4i − 1.0 (− 3.0/0.0) pt, than in the group receiving oral Semaglutide 0.0 (− 1.0/1.0) pt, *p* < 0.0001.

### Logistic regression analysis

Univariate logistic regression analysis showed statistically significant associations between oral semaglutide therapy, variation in hs-CRP levels, and changes in BMI from baseline to follow-up with the incidence of cognitive impairment (CoI) (Supplementary Table S2).

In a multivariate stepwise regression model oral semaglutide therapy was associated with a 77% reduction in the incidence of CoI (OR 0.231, 95% CI 0.113—0.474; *p* < 0.0001). The variation in hs-CRP levels between baseline and follow-up was associated with a 47% reduction in CoI incidence (OR 0.539, 95% CI 0.337–0.862; *p* = 0.010). Notably, a change in BMI from baseline to follow-up was associated with a 14% reduction in the risk of CoI incidence in overall the study population (OR 0.865, 95% CI 0.760–0.984; *p* = 0.027) (Table 3).

**Table 3:** Multivariate stepwise logistic regression analysis on CoI incidence in the whole study population

	OR	CI 95%	*p*
Oral Semaglutide, yes/no	0.231	0.113—0.474	<0.0001
Δ hs-CRP, mg/l	0.539	0.337—0.862	0.010
Δ BMI, Kg/m^2^	0.865	0.760—0.984	0.027

*Δ* variation between baseline and 1-year follow-up, *hs-CRP* high sensitivity C reactive protein, *BMI* body mass index

## Discussion

The present study demonstrates that in elderly patients with HFpEF, obesity, and type 2 diabetes who were already on SGLT2i therapy, oral semaglutide was associated with a reduced incidence of cognitive impairment over a one-year follow-up period compared to DPP4i.

The mechanisms by which oral semaglutide therapy may reduce the risk of CoI in the elderly are likely multifactorial. Firstly, the superior and more durable glycometabolic control achieved with oral semaglutide compared to DPP4i is likely to be an important protective mechanism. In fact, our study observed a greater and more sustained reduction in HbA1c levels and HOMA in the oral semaglutide group versus the DPP4i group. This was accompanied by a more pronounced reduction in BMI in patients receiving oral semaglutide (see Table 2).

Moreover, micro- and macrovascular complications of type 2 diabetes are important contributors to CoI, especially in the elderly and diabetic populations. In type 2 diabetes patients, especially during periods of glycometabolic decompensation when there is an increase in systemic inflammation and reactive oxygen species (ROS) production. These processes alongside endothelial dysfunction are associated with CoI incidence [26, 27]. Additionally, insulin resistance is linked to increased ROS production, including in the brain. The insulin-degrading enzyme (IDE) may also play a role in the pathophysiological mechanisms underlying CoI. In conditions of insulin resistance, reduced IDE activity or availability leads to impaired degradation of β-amyloid, which competes with insulin for IDE binding. This results in β-amyloid accumulation and the formation of amyloid plaques, further contributing to oxidative stress, neuronal damage, and neurodegeneration. Alterations in the blood–brain barrier and the GABAergic pathway are also associated with type 2 diabetes and may contribute to CoI [21].

There is emerging evidence that GLP1-RAs can counteract these pathophysiological mechanisms. Studies in both mouse models and humans have shown that GLP1-RAs, such as liraglutide, can counteract insulin receptor downregulation and synapse damage by activating cAMP/PKA signaling, thereby improving cognitive function in mice [28]. Another study demonstrated that liraglutide reversed cognitive impairment and prevented ultrastructural neuronal damage in a mouse model of Alzheimer's disease. This neuroprotective effect is likely due to its ability to reduce tau phosphorylation in the brain via modulation of the PI3K/Akt/GSK-3β pathway [29].

In humans, promising results have also been observed. A nested case–control study reported that the use of GLP1-RAs significantly reduced the risk of dementia in individuals with type 2 diabetes [30]. Similar benefits were seen in obese patients with type 2 diabetes and CoI, where 3-month therapy with GLP1-RAs led to enhanced activation of the right hippocampus and improved cognitive function [31]. Furthermore, a double-blind, placebo-controlled clinical trial in patients with Alzheimer’s disease found that liraglutide increased brain glucose metabolism, delaying the development of cognitive impairments over a 26-week follow-up. However, in this study, GLP1-RAs had no effect on β-amyloid clearance [32].

It is important to note that the aforementioned studies primarily focused on diabetic and/or obese patients, not specifically on elderly patients with HFpEF, as in the present study. Furthermore, these studies had relatively short follow-up periods, which did not extend to one year.

In our study, we also observed a significant reduction in circulating levels of high-sensitivity C-reactive protein (hs-CRP), which was notably greater in the oral semaglutide group compared to the DPP4i group. This reduction in hs-CRP levels was associated with a 47% reduction in the risk of CoI. These findings are in keeping with sub-analyses of the STEP-1, STEP-2, and STEP-3 trials, which demonstrated that injectable semaglutide therapy led to a greater reduction in CRP levels compared to placebo, regardless of baseline glycemic status or BMI. These trials suggested an anti-inflammatory effect of semaglutide therapy in obese individuals [33].

Our findings are also consistent with results from other studies that have demonstrated a strong association between GLP1-RA therapy and reduced circulating levels of inflammatory markers. The anti-inflammatory effect appears to contribute not only to cardiovascular protection but also to a decreased risk of CoI. A recent meta-analysis of 40 randomized controlled trials demonstrated that GLP1-RA treatment significantly reduced CRP levels compared to placebo, and this reduction correlated with improvements in systolic blood pressure and insulin sensitivity [34]. However, the meta-analysis included diabetic patients without HFpEF, with a lower mean age compared to our study population, and a follow-up duration of only six months.

Our study, conducted in elderly patients with type 2 diabetes, obesity, and HFpEF, demonstrated an association between oral semaglutide therapy and improved inflammatory and metabolic profiles compared to DPP4i. Specifically, despite being a retrospective observational study, it represents the first study to demonstrate a significant association between oral semaglutide therapy and a reduced risk of CoI at one-year follow-up in patients on stable hypoglycaemic therapy, including SGLT2i for at least six months.

This study does have several limitations. Firstly, it is not a randomized clinical trial. Another important limitation is the relatively small sample size. However, this study enrolled patients who are often underrepresented in large randomized controlled trials like multi-morbid elderly diabetic patients with HFpEF.

## Conclusion

This real-life study of a cohort of multi-morbid elderly patients with chronic HFpEF, obesity, type 2 diabetes, demonstrated that treatment with oral semaglutide for one year was associated with a 77% reduction in the risk of incident CoI. Semaglutide-induced reductions in circulating levels of hs-CRP and BMI were linked to the reduction in the risk of CoI. Our findings confirm the multi-systemic protective role and anti-inflammatory effects of oral semaglutide in a cohort of multimorbid elderly patients with type 2 diabetes, obesity, and HFpEF, who were already on stable background therapy with SGLT2i.

## Supplementary Information

Below is the link to the electronic supplementary material.

Supplementary file1 (DOCX 28 KB)

## Data Availability

The raw data supporting the conclusions of this article will be made available by the authors, without undue reservation.
