# Supplementary material for: Oral semaglutide vs DPP-4 inhibitors in reducing risk of cognitive impairment in elderly patients with HFpEF and obesity
Source: Intern Emerg Med. 2026 Aug 20;21(8):2941–9. doi: 10.1007/s11739-026-04499-4 (PMC13751725; doi:10.1007/s11739-026-04499-4)
Supplement: Supplementary file 1 [file 11739_2026_4499_MOESM1_ESM.docx]

**SUPPLEMENTARY TABLES**

**Supplementary table 1. Clinical, epidemiological, laboratory parameters of study participants at baseline, according to therapy with oral semaglutide or DPP4i.**

|  | **All population (n. 246)** | **Oral semaglutide  (n. 123)** | **DPP4i (n. 123)** | **p*** |
| --- | --- | --- | --- | --- |
| **SBP,** *mmHg* | 139.6±4.97 | 139.7±3.6 | 139.6±6.0 | 0.848**^ǂ^** |
| **DBP,** *mmHg* | 84.1±2.98 | 83.7±2.5 | 84.5±3.4 | 0.032**^ǂ^** |
| **Pulse Pressure,** *mmHg* | 55.5±5.97 | 55.9±4.4 | 55.0±7.2 | 0.220**^ǂ^** |
| **HR,** *bfm* | 70.33±9.7 | 69.7±10.1 | 70.99±9.33 | 0.283**^ǂ^** |
| **Hb,** *g/dl* | 14.1±0.6 | 14.1±0.4 | 14.1±0.8 | 0.370**^ǂ^** |
| **Hct,** *%* | 43.2±3.1 | 43.5±2.2 | 42.9±3.7 | 0.112**^ǂ^** |
| **PLT,** **10^^3^/mm^3^* | 254±56 | 254±56 | 255±56 | 0.927**^ǂ^** |
| **Na,** *mmol/l* | 141.2±2.6 | 141.6±2.4 | 140.9±2.8 | 0.064**^ǂ^** |
| **K,** *mmol/l* | 4.6±0.25 | 4.6±0.2 | 4.5±0.3 | 0.002**^ǂ^** |
| **HOMA,** *pts* | 7.81±0.90 | 7.9±0.8 | 7.7±0.9 | 0.069**^ǂ^** |
| **HbA1c,** *%* | 8.1±0.6 | 8.1±0.7 | 8.2±0.6 | 0.348**^ǂ^** |
| **Glicemia,** *mg/dl* | 141.6±8.3 | 142.2±7.8 | 141.0±8.7 | 0.300**^ǂ^** |
| **Insulinemia,** *mU/L* | 22.32±2.20 | 22.53±2.0 | 22.11±2.4 | 0.141**^ǂ^** |
| **Creatinina,** *mg/dl* | 1.12±0.12 | 1.07±0.13 | 1.2±0.08 | <0.0001**^ǂ^** |
| **eGFR,** *ml/min/1.73m^2^* | 61.6±13.8 | 68.4±13.6 | 54.7±10.2 | <0.0001**^ǂ^** |
| **Total Colesterol,** *mg/dl* | 162.1±11.5 | 162.2±11.5 | 162.0±11.7 | 0.865**^ǂ^** |
| **LDL,** *mg/dl* | 91.3±11.9 | 90.9±12.2 | 91.7±11.7 | 0.608**^ǂ^** |
| **HDL,** *mg/dl* | 39.7±3.1 | 40.3±3.3 | 39.0±2.8 | 0.002**^ǂ^** |
| **Triglycerides,** *mg/dl* | 155.6±8.5 | 155.2±9.2 | 156.2±7.8 | 0.359**^ǂ^** |
| **AST,** *U/L* | 26.5±5.8 | 26.2±5.8 | 26.7±5.7 | 0.508**^ǂ^** |
| **ALT,** *U/L* | 32.7±4.0 | 32.4±4.0 | 33.0±4.0 | 0.213**^ǂ^** |
| **γ-GT,** *U/L* | 44.9±8.5 | 44.5±9.2 | 45.3±7.7 | 0.457**^ǂ^** |
| **Total Bilirubin,** *mg/dl* | 0.53±0.16 | 0.52±0.15 | 0.55±0.16 | 0.078**^ǂ^** |
| **Alkaline Phosphatase,** *U/L* | 91.6±7.8 | 91.9±7.5 | 91.3±8.1 | 0.541**^ǂ^** |
| **Albumin,** *mg/dl* | 4.3±0.44 | 4.2±0.5 | 4.4±0.4 | 0.002**^ǂ^** |
| **NT-pro-BNP,** *pg/ml* | 837.1±162.6 | 841.35±183.4 | 832.9±139.3 | 0.686**^ǂ^** |
| **Uric acid,** *mg/dl* | 6.7±0.2 | 6.7±0.2 | 6.7±0.2 | 0.455**^ǂ^** |
| **hs-CRP,** *mg/l* | 4.1±0.6 | 4.1±0.5 | 4.1±0.6 | 0.797**^ǂ^** |

*** performed by χ-square test**

**^ǂ^ performed by t-test for unpaired data**

**Abbreviations:** **DDP4i:** Dipeptidyl peptidase-4 inhibitors; **BMI:** Body mass index; **IHD:** ischemic heart disease; **AF:** atrial fibrillation; **SAS:** sleep apnea syndrome; **CKD:** chronic kidney disease; **COPD:** Chronic obstructive pulmonary disease; **NAFLD:** Non-alcoholic fatty liver diasease; **T2DM:** type 2 diabetes mellitus; **MLHFQ:** Minnesota Living with Heart Failure Questionnaire; **MNA:** Mini-nutritional assessment; **SBP:** Systolic blood pressure, **DBP**: Diastolic blood pressure; **HR:** heart rate; **RR:** respiratory rate; **Hb:** haemoglobin; **Hct**: haematocrit, **Na:** Sodium; **Mg:** Magnesium; **K:** Potassium; **HOMA:** Homeostasis model assessment; **HbA1c:** glycated haemoglobin; **e-GFR:** estimate glomerular filtration rate; **LDL:** low density lipoproteins; **AST:** aspartate aminotransferase; **ALT:** alanine aminotransferase; **γ-GT:** gamma-glutamyltransferase; **NT-pro-BNP:** N-terminal pro-brain natriuretic peptide; **hs-CRP:** high sensitivity C reactive protein;

**Supplementary table 2. Univariate logistic regression analysis on CoI incidence in the whole study population.**

|  | **OR** | **95% CI** | **p** |
| --- | --- | --- | --- |
| **Age,** *10 years* | 0.877 | 0.589 – 3.040 | 0.516 |
| **Female sex,** *yes/no* | 0.783 | 0.400 – 1.536 | 0.477 |
| **Smokers,** *yes/no* | 2.200 | 1.013 – 4.779 | 0.046 |
| **Δ BMI,** *Kg/m^2^* | 0.852 | 0.744 – 0.977 | 0.022 |
| **SAS,** *yes/no* | 1.441 | 0.747 – 2.782 | 0.276 |
| **CKD,** *yes/no* | 1.441 | 0.874 – 3.318 | 0.118 |
| **β-blockers,** *yes/no* | 2.648 | 1.316 – 5.329 | 0.006 |
| **Anti-platelets drugs,** *yes/no* | 1.153 | 0.618 – 2.152 | 0.654 |
| **PCSK9i,** *yes/no* | 1.509 | 0.749 – 3.040 | 0.250 |
| **LVEF,** *%* | 1.120 | 0.979 – 1.282 | 0.100 |
| **Oral Semaglutide,** *yes/no* | 0.340 | 0.115 – 0.828 | 0.032 |
| **Δ KCCQ-CCS,** *pt* | 0.990 | 0.953 – 1.028 | 0.600 |
| **Δ HOMA,** *pt* | 0.959 | 0.666 – 1.381 | 0.821 |
| **Δ HbA1c,** *%* | 1.273 | 0.745 – 2.174 | 0.377 |
| **Δ hs-CRP,** *mg/l* | 0.494 | 0.295 – 0.827 | 0.007 |
| **Serum albumin,** *g/dl* | 1.426 | 0.688 – 2.958 | 0.340 |

**Abbreviations: Δ:** variation between baseline and 1-year follow-up; **BMI:** body mass index; **SAS:** sleep apnea syndrome; **CKD:** chronic kidney disease; **LVEF:** Left ventricular ejection fraction; **DPP4i:** Dipeptidyl peptidase-4 inhibitors; **KCCQ-CSS:** Kansas City Cardiomyopathy Questionnaire- clinical summary score; **HOMA:** Homeostasis model assessment; **hs-CRP:** high sensitivity C reactive protein.
